# Expression of prokineticin-receptor2(PK-R2) is a new prognostic factor in human colorectal cancer

**DOI:** 10.18632/oncotarget.5565

**Published:** 2015-09-10

**Authors:** Takanori Goi, Hidetaka Kurebayashi, Yuki Ueda, Takayuki Naruse, Toshiyuki Nakazawa, Kenji Koneri, Yasuo Hirono, Kanji Katayama, Akio Yamaguchi

**Affiliations:** ^1^ First Department of Surgery, University of Fukui, Japan

**Keywords:** prokineticin-receptor2, colorectal cancer, prognostic factor

## Abstract

The increased invasiveness of colorectal cancer cells is important for progression and metastasis to the surrounding organs. According to recent molecular biological studies, signaling through transmembrane Prokineticin-Receptor2(PK-R2) is likely involved in the ability of tumor cell to invade. However, no studies have evaluated the relationship between PK-R2 expression, ability of cancer to invade/metastasize, and patient prognosis in cases of resected colorectal cancer. Accordingly, we have examined these factors in the present study.

Immunohistochemical staining was performed to detect PK-R2 in the primary lesion and adjacent normal large intestine mucosa of 324 colorectal cancer patients who underwent resection surgery at our department. Additionally, we conducted clinicopathologic examinations and analyzed patient prognoses with the Kaplan-Meier method. Further, multivariate analysis was conducted using a cox-proportional hazard model.

PK-R2 expression was observed on the cellular membrane of the primary lesion in 147 of 324 cases (45.3%) of human colorectal cancer. PK-R2 expression was associated with a higher incidence of vascular invasion, lymph node metastasis, hepatic metastasis, and hematogenous metastasis. Further, prevalence of PK-R2 expression increased as tumor stage increased. In stage III curative resection cases, where recurrence is the most serious problem, cases that expressed PK-R2 had a significantly lower 5-year survival rate (82.1% versus 66.8%) and higher recurrence compared to those cases with no PK-R2 expression. In the multivariate analysis for prognosis, PK-R2 expression was found to be an independent factor(ratio2.621).

PK-R2 expression could be one of the new prognostic factors in human colorectal cancer.

## INTRODUCTION

Colorectal cancer has one of the highest prevalence and mortality rates among malignant tumors [1–4]. Hematogenous metastasis of colorectal cancer to other organs including the liver and lung is particularly frequent, driving the need for therapies that minimize the cancer spread [5, 6]. One model for the mechanism of hematogenous metastasis of colorectal cancer involves growth of tumor at the primary lesion, dissociation of cancer cells, invasion into interstitium, invasion into blood vessels, and implantation and growth in the liver and other distant organs. Meanwhile, various genetic alterations have been studied, and involvement of a number of genes has been reported [7–10].

The invasiveness of colorectal cancer is influenced by the interaction between the cell surface proteins of the tumor cell and the extracellular matrix. Proteases such as matrix metalloprotease (MMP) break down the extracellular matrix to allow the tumor cell to pass through the base membrane and penetrate the inside of the tissue [11, 12]. Fibronectin, a component of the extracellular matrix, is broken down into fragments, accelerating the expression of the protease gene, and supplying the cell growth-promoting factor, thus strengthening the ability of the cells to proliferate [13–15]. Further, the transition of cancer cells to a fibroblast-like mesenchymal state (epithelial-mesenchymal transition; EMT) and the transition from a mesenchymal state to an amoeboid state (mesenchymal-amoeboid transition; MAT), are known to play an important role in cancer cell invasiveness [16–18]. The involvement of the Rho GTPase family in this mechanism has been observed, and drugs targeting these proteins have recently been developed [19, 20].

Prokineticin-Receptor2(PK-R2) is a known ligand of Prokineticin1(PROK1) and transduces important biological signals to induce physiological changes [21–23]. The PROK1/EG-VEGF protein was identified by Ferrara as a vascular endothelial growth factor expressed only in limited tissues including healthy endocrine tissue [24]. Recently, its association with malignant tumors has been studied, and the following findings have been obtained: 1) increased PROK1 expression is associated with increased malignancy of prostate cancer, neuroblastoma, thyroid cancer and pancreatic duct cancer [25–28]; 2) PROK1 mRNA expression in the resected primary lesion of colorectal cancer patients is associated with a significantly worse prognosis, compared with those patients who did not express PROK1 [29–32]; and 3) increased PROK1 expression is associated with angiogenesis involving hepatic metastasis [33]. Further, cellular invasion is associated with the autocrine signaling mechanism of PROK1/PK-R2.

The present study was conducted to compare the expression of PK-R2 protein with the invasiveness of the primary lesion in human colorectal cancer, as well as with the clinicopathologic features and patient prognoses, as reported below.

## RESULTS

### PK-R2 expression in human colorectal cancer

**Figure 1:**
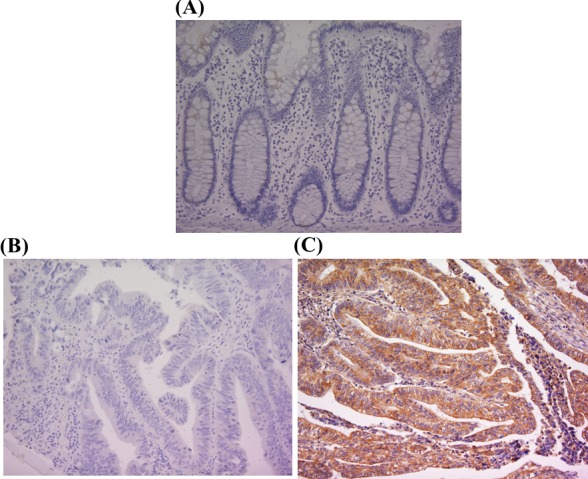
PK-R2 protein expression in healthy human colorectal mucosa and human primary colorectal cancer by immunohistochemical staining with anti-PK-R2 mAb **A.** PK-R2 expression was not detected in healthy human colorectal mucosa. **B.** PK-R2 expression was not detected in the cell membrane, the cytoplasm, and the nucleus of primary colorectal cancer lesions. **C.** PK-R2 protein was stained in brown. PK-R2 protein was detected in both the cell membrane and the cytoplasm of primary colorectal cancer lesions. The intensity was stronger in the cell membrane than in the cytoplasm.

While PK-R2 expression was not observed in the healthy mucosal membrane adjacent to human colorectal cancer, its expression was found in the primary lesion of colorectal cancer. Figure 1 shows a representative case. PK-R2 expression was observed on the cellular membrane. PK-R2 expression was observed in 147 (45.3%) of 324 colorectal cancer patients.

### PK-R2 expression and clinicopathologic factors in human colorectal cancer tissue

No relationships were detected between PK-R2 expression and the following clinicopathologic factors: gender, age, location, histological type, lymphatic invasion, venous invasion, depth invasion, or lymph node metastasis. However, expression was significantly higher in the cases with peritoneal dissemination, hematogenous metastasis, and TNM stage (Table 1).

**Table 1:** Correlation Between Clinicopathologic Findings and PK-R2 Expression

	No of cases	PK-R2 positive		P-value
		No of cases	%	
All cases	324	147 (45.3%)		
Gender				p=0.549
Male	180	79 (43.8%)		
Female	144	68 (47.2%)		
Age (average 66.5)				p=0.119
<55	55	32 (58.1%)		
55-64	71	33 (46.4%)		
65-74	98	44 (44.8%)		
≧75	100	38 (38.0%)		
Location				P=0.108
Right colon	122	49 (40.1%)		
Left colon	112	49(43.7%)		
Rectum	90	49 (54.4%)		
Histological type				p=0.956
Well+Mode	299	135 (45.1%)		
Poor	19	9 (47.3%)		
Mucinous	6	3 (50.0%)		
Lymphatic invasion				p=0.893
Negative	41	19 (46.3%)		
Positive	283	128 (45.2%)		
Venous invasion				p=0.365
Negative	89	44 (49.4%)		
Positive	235	103 (43.8%)		
Peritoneal metastasis				p=0.047
Negative	315	140 (44.4%)		
Positive	9	7 (77.7%)		
Hematogenous Metastasis		p=0.029		
Negative	286	114 (39.9%)		
Positive	38	33 (86.8%)		
T(TNM 6th)				p=0.133
T1, T2	147	60 (40.8%)		
T3, T4	177	87 (49.5%)		
N(TNM 6th)				p=0.323
N0	153	65 (42.4%)		
N1, N2	171	82 (47.9%)		
Stage(TNM 6th)				p<0.001
I	42	13 (30.0%)		
II	104	47 (45.1%)		
III	135	50 (37.4%)		
IV	43	37(86.0%)		

Well, well differentiated adenocarcinoma; Mode, moderately differentiated adenocarcinoma; Poor, poorly differentiated adenocarcinoma; Muc, mucinous adenocarcinoma.

### Relationship between PK-R2 expression and the stage of colorectal cancer

PK-R2 expression was found in 13 (30%) of 42 Stage I colorectal cancer patients, 47 (45.1%) of 104 Stage II patients, 50 (37.5%) of 135 Stage III patients, and 37 (86%) of 43 Stage IV patients, indicating that the expression rate went up with the advancement of stage(Table 1).

### Relationship between PK-R2 expression and survival rate in all colorectal cancer patients

**Figure 2:**
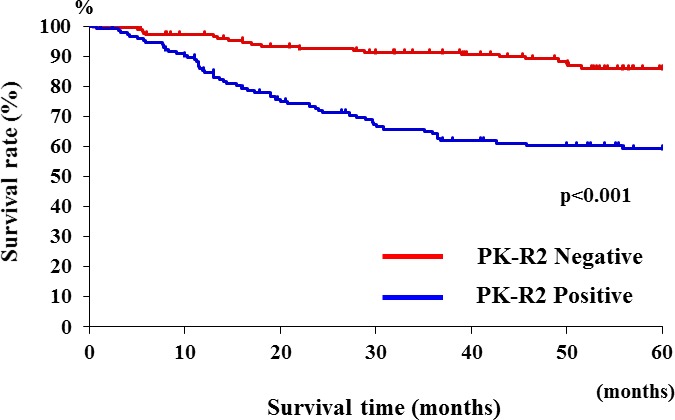
Relationship between PK-R2 expression and survival rates in all human colorectal cancer patients Patients with PK-R2-positive tumors had significantly poorer prognosis than those with PK-R2-negative tumors(*p* < 0.001).

The 5-year survival rate was 85.9% in the colorectal cancer patients with no PK-R2 expression in their primary lesion, whereas the 5-year survival rate of patients with PK-R2 expression was significantly lower (59.3%)(Figure 2).

### Relationship between PK-R2 expression and recurrence rate by stage of colorectal cancer

The tumor recurrence rate among patients with Stage III colorectal cancer was significantly higher for patients with PK-R2 expression in their primary lesion compared to patients who did not express PK-R2 (36% vs. 20%, respectively)(Table 2). Among patients with Stage I and Stage II colorectal cancers, no significant difference was observed in the recurrence rate between patients who expressed PK-R2 in their primary lesion and those that did not.

**Table 2:** Relationship between *PK-R2* expression and the recurrence rate of metastasis by colorectal cancer stage

	PK-R2 negative			PK-R2 positive			P
Stage							
Grouping	No of cases	recurrence	%	No of cases	recurrence	%	
All cases	171	22	12.9%	110	27	24.5%	0.011
I	29	0	0%	13	0	0%	
II	57	5*	8.8%	47	9**	19.1%	0.122
III	85	17***	20.0%	50	18****	36.0%	0.011

(*Hematogenous metastasis 3cases, Peritoneal dissemination 2cases, **Hematogenous metastasis 4cases, Peritoneal dissemination 5cases, ***Hematogenous metastasis 13cases, Peritoneal dissemination 2cases, Lymphnode metastasis 4cases, ****Hematogenous metastasis 9cases, Peritoneal dissemination 5cases, Lymphnode metastasis 4cases)

### Relationship between PK-R2 expression and survival rate by colorectal cancer stage

**Figure 3:**
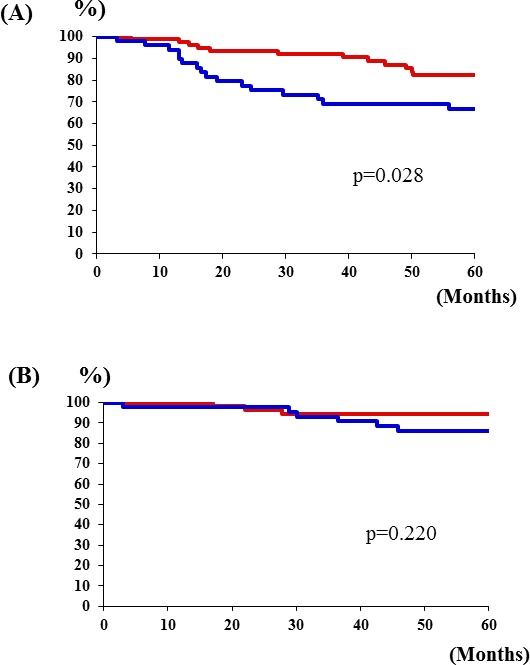
Relationship between PK-R2 expression rates and survival rates by stage of human colorectal cancer patients **A.** Stage III: Patients with PK-R2-positive tumors had significantly poorer prognosis than those with PK-R2-negative tumors in Stage III(*p* = 0.028). **B.** No significant differences in survival were observed between patients with Stage II colorectal cancer in terms of PK-R2 expression in the primary tumors(*p* = 0.220).

The 5-year survival rate for Stage III colorectal cancer patients with PK-R2 expression-negative primary tumors was 82.1%, whereas it was 66.8% for patients with PK-R2 expression-positive tumors (*p* = 0.028) (Figure 3A). No significant differences in survival were observed between patients with Stage I, II and Stage IV colorectal cancer in terms of PK-R2 expression in the primary tumors.

### Clinicopathologic prognostic factors based on multivariate analysis

The factors found to differ significantly between PK-R2-expressing and non-expressing patients by univariate analysis were examined by multivariate analysis. histological type, lymphatic invasion, peritoneal metastasis, hematogenous metastasis, and PK-R2 expression were determined to be clinicopathologic prognostic factors. The risk rate for PK-R2 expression was 2.621(Table 3).

**Table 3:** Pathological Findings and PK-R2 as Prognostic Factor for Colorectal Cancer Patients

	Univariate analysis			Multivariate analysis
	Hazard Ratio	95% CI	P-value		Hazard Ratio	95% CI	P-value
Gender	0.696	0.436-1.109	0.127			
Age	0.785	0.635-0.970	0.025	0.840	0.673-1.047	0.121
PK-R2	4.798	2.939-7.834	<0.001	2.621	1.519-4.525	0.001
Histological type	1.999	1.376-2.930	<0.001	2.134	1.406-3.241	<0.001
(Well^a^+Mode^2^ /Poor^c^ / Muc^d^)						
Serosal invasion	2.652	1.591-4.421	<0.001	1.632	0.936-2.844	0.084
Lymphatic invasion	5.106	3.059-8.522	<0.001	2.450	1.210-4.961	0.013
Venous invasion	4.860	3.056-7.729	<0.001	1.082	0.599-1.955	0.795
Lymphnode metastasis	4.003	2.305-6.954	<0.001	1.414	0.697-2.868	0.338
Peritoneal metastasis	19.863	9.028-43.704	<0.001	5.119	2.150-12.150	<0.001
Hematogenous	16.947	10.376-27.679	<0.001	5.336	2.914-9.773	<0.001
Metastasis						

a Well, well differentiated adenocarcinoma

b Mode, moderately differentiated adenocarcinoma

c Poor, poorly differentiated adenocarcinoma

d Muc, mucinous adenocarcinoma.

## DISCUSSION

The greatest risk factor for mortality among malignant tumors is metastasis [1–4], and the study of such an important process may lead to new cancer therapies. Several reports describe the factors related to the invasiveness of malignant tumors and patient prognosis. Specifically, MMP, a protease that degrades the extracellular matrix, and the processes of EMT and MAT have been studied [11, 12, 16–18]. The PK-R2 factor examined in the present study is positioned on chromosome 20p13. It is a G protein-coupled receptor that is present on the cell surface, with the role of receptor factor [21–23]. Members of the prokineticin family act as ligands to this receptor and have diverse biological functions. PROK2, a member of the prokineticin family, is positioned on chromosome 3p21.1, and is associated with Kallmann syndrome and hypogonadotropic hypogonadism with normal olfactory function [34–40]. Meanwhile, PROK1, positioned on chromosome 1p21, was reported to act as a vascular endothelial growth factor in the endocrine cell [24]. Subsequently, PROK1 was found to be strongly expressed in a number of malignant tumors including prostate cancer, neuroblastoma, pancreatic duct cancer, thyroid cancer, and colorectal cancer, and a relationship with the level of malignancy was confirmed [25–32]. We further found that tumor growth, hepatic metastasis, and angiogenesis were induced when PROK1 was strongly expressed in a low-PROK1 expressing colorectal cancer cell strain. It was recently found that PK-R2, a receptor for PROK1, was expressed in the same colorectal cancer cell strain, playing a role in cellular invasion via autocrine signaling induced by PROK1. This receptor is considered to influence various metastatic mechanisms. As no reports are currently available on the expression of PK-R2 in the primary lesion of resected human colorectal cancer and the relationship of that expression with patient prognosis, we undertook the present study.

PK-R2 expression was found in the primary lesion of 40% of human colorectal cancer resection cases. In the PK-R2-expression cases, the prevalence of clinicopathologic events was high, such as iHstological type, peritoneal metastasis, and hematogenous metastasis, and associated with poor patient prognosis. PK-R2 expression was also identified as an independent prognostic factor. Based on these findings, PK-R2 expression may be an important factor in the mechanism of metastasis, initiating dissociation of cancer cell from the primary lesion, followed by disintegration of basement membrane, migration within interstitium, and vascular invasion of the tumor cell.

In the present study, PK-R2 expression in the primary lesion of human colorectal cancer was found to be a new prognostic factor for colorectal cancer, because of its involvement in the invasiveness of tumor.

## MATERIALS AND METHODS

### Patients and samples

Surgical specimens and adjacent normal colorectal tissues were obtained from surgical resection from 324 patients with sporadic primary colorectal cancer in the First Department of Surgery, University of Fukui, Japan between 1990 and 2007. The age of the 324 patients ranged from 22 to 95 years. Cancerous tissues and corresponding normal tissues were obtained at surgery. According to the TMN classification [41], 42, 104, 135, and 43 were I, II, III, and IV respectively. As histopathological findings varied within the same tumors, the diagnosis was based upon the dominant pattern evaluated by two pathologists. All sample were fixed in 10% paraformaldehyde(pH6.8) for 24 h, and embedded in paraffin.

The eligibility criteria were as follows: (1) a histopathological findings confirmed primary colorectal cancer, (2) resection of colorectal cancer with extended (D2 or D3) lymph node dissection [42], (3) histological curative resection(StageI∼III), (4) an Eastern Cooperative Oncology Group performance status(PS) of 0 or 1, (5) no chemotherapy or radiotherapy before surgical resection, (6) Patients with stage III received tegafur-uracil-based chemotherapy after surgical resection, (7) Patients with stage IV received 5-fluorouracil-based after surgical resection, (8) Patients with stage I/II received no chemotherapy after surgical resection, (9) All patients were followed up for recurrence at regular intervals for five years, underwent chest X-ray, computed tomography, and colonoscopy.

### Immunohistochemical study

Paraffin sections, 4μm thick, were de-paraffinized with xylene and dehydrate through a graded ethanol series. Endogenous peroxidase activity was blocked by incubation for 30 minutes with 1% hydrogen peroxidase in methanol. These hydrate sections were incubated in a dilution of normal goat serum for 20 minutes to reduce nonspecific staining, and incubated with anti-PK-R2 Ab(Novus Biochemicals, CO, USA) for 1 hour. After washing with Phosphate Buffered Saline(PBS) buffer, and analyzed for the expression of PK-R2 protein by the ChemMate method(Dako, Denmark) [32]. Finally, the slides were lightly counterstained with hematoxylin. The expression was interpreted as positive when the protein was expressed in more than 10% of all cancer cells using Image J Software (http://rsb.info.nih.gov/ij/).

### Statistical analysis

The association of PK-R2 expression with clinicopathological findings was assessed by cross-tabulation, and statistical evaluations were determined by the χ^2^ test using Stat Mate IV(ATMS Co., Ltd., Japan).

Patient survival was calculated using the Kaplan-Meier technique. The outcomes from different groups of patients were compared by log rank test using Stat Mate IV(ATMS Co., Ltd., Japan).

The Cox proportional hazards model was used in multivariate regression analyses of survival date using SPSS soft ware(IBMM SPSS Statistics, IBM Corporation, USA).

*P* values < 0.05 were considered statistically significant.
